# Customer demand-driven low-carbon vehicles combined strategy and route optimisation integrated decision

**DOI:** 10.1038/s41598-021-98028-2

**Published:** 2021-09-16

**Authors:** Hanwen Liu, Xiaobing Liu, Sardar M. N. Islam, Xueqiao Yu, Qiqi Miao, Yapin Chen, Lin Lin

**Affiliations:** 1 School of Economics and Management, Dalian University of Technology, Dalian, 116024 China; 2 ISILC, Victoria University, Melbourne, 80309 Australia; 3 Transportation & Economics Research Institute, China Academy of Railway Sciences Corporate Limited, Beijing, 100080 China; 4 Faculty of Geography, Yunnan Normal University, Kunming, 650050 China; 5 CRRC Dalian R&D Co., Ltd., Lvshun, 116052 China

**Keywords:** Engineering, Mathematics and computing

## Abstract

With the optimal operating cost and optimal carbon emission target of the chemical logistics companies, a low-carbon routing optimisation with a multi-energy type vehicle combined problem is proposed by considering the concept of the logistics companies’ low-carbon behaviour. An integrated decision-making of multi-energy type vehicles combined strategy and route optimisation based on customer demand is presented, and an improved genetic algorithm is designed. A case study is then applied based on the data collected from the case research. The effectiveness of the improved genetic algorithm is tested. The two joint objectives of operating cost and carbon emission are examined through the cost analysis of environmental energy vehicles and traditional energy vehicles in different combination scenarios. The case analysis shows that a rational multi-energy type vehicle combination with route optimisation has a significant correlation with the operating cost and carbon emissions, while the environmental vehicle purchasing cost reduction and subsidy policy affect the operating cost.

## Introduction

### Background

From the perspective of lean logistics management, the transportation equipment system is planned to achieve cost reduction and increased efficiency, energy savings and emission reduction^1^. The means of transportation equipment mentioned in this paper is a drop trailer vehicle. This paper discusses the impact of chemical logistics companies’ operating costs and carbon emission factors on transportation equipment resource allocation decision-making. For chemical logistics companies, transportation equipment is an indispensable part of operating resources. Carbon emissions mainly come from the exhaust emissions generated by traditional energy transportation equipment during the transportation process^2^. Therefore, chemical logistics companies should pay attention to environmentally friendly transportation equipment. A reasonable environmental transportation equipment purchase plan formed a multi-energy type of transportation fleet, which has become an essential part of the rational allocation of resources to achieve lean logistics management.

Chemical logistics companies are responding to the increasingly severe global greenhouse effect^3^ and the data reported by the ‘Carbon Tracker’ report^4^. Claims that have demanded fossil fuels peak between 2020 and 2030 and have promoted logistics companies to engage in proactive low carbon behaviour^5^. This behaviour shows that to achieve energy savings and carbon emission reduction and to protect the environment, chemical logistics companies are willing to systematically plan the configuration of existing transportation equipment to reduce carbon emissions.

Customer demand is the power source that drives logistics companies’ operation and the foundation for companies to realise value. Moreover, customer demand is the most intuitive expression of profitability, and it is also one of the cores for companies to provide lean logistics services^6^. Based on customer demand, chemical logistics companies should comprehensively consider sustainable profitability and transportation network planning to upgrade their existing transportation equipment. The purpose is to address the possible future fossil fuel energy crisis and the impact of “carbon policy” on chemical logistics companies. Simultaneously, the companies’ “low-carbon” label will bring huge brand profits^7^, and give companies more advantages in differentiated competition.

Two aspects should be considered when making decisions to upgrade transportation equipment based on the balance of cost and carbon emissions. First, customer demand is the value foundation of chemical logistics companies. The purchase of environmentally friendly transportation equipment based on customer demand can better control the operating cost. Furthermore, the company’s plan should be finding the optimal value of the company’s operating cost and using this as a necessary parameter to make the plan. Second, the upgrade of transportation equipment should be comprehensive, as the transportation equipment purchase decision made by vehicle static cost assessment does not fully meet the goal of the company’s operating cost control and carbon emission reduction. The dynamic carbon emission indicators of transport equipment with heavier loads should also be taken into consideration. The total carbon emissions generated by transportation equipment should be calculated based on customer demand in a transportation network with the actual load. The cost of carbon emissions should be included in the operating cost as a parameter. In summary, combining these two aspects to formulate a comprehensive decision-making plan could achieve the sustainable development strategic goal of chemical logistics companies: it can not only achieve the environmental protection objective but also ensure that the purchase plan does not affect the company’s sustainable operations.

In the existing cost and carbon emissions research, Choi et al. selected several different energy type vehicles and analysed them on a well-to-wheel basis. Six constructed programs were made based on the different energy policies. They calculated the life cycle of greenhouse gas emissions of electricity and hydrogen corresponding to the energy policies to evaluate the advantages of using different energy type vehicles^8^. Cox et al. compared the current (2017) and future (2040) vehicle life cycle environmental burdens and total cost of ownership (TCO) for different powertrain configurations, performing a global sensitivity analysis based on Monte Carlo to determine that the vehicles powertrain's electrification as an effective way to reduce carbon emissions^9^. Gružauskas et al. used a visual logistics network model, combined with digital transformation strategies, leading to a proposed logistic clusters and shared transportation to reduce logistics network costs and carbon emissions^10^. Based on the idea of vehicle life cycle analysis, Ma compared the user costs of electric vehicles and traditional logistic vehicles and used vehicle cost sensitivity analysis to draw vehicle selection conclusions^11^. Nie et al. predicted the cargo turnover in major cities based on the improved Gompertz model. The predicted turnover compared the total carbon emissions under environmental energy vehicles conditions with different capacities. They believed that the continuous innovation of environmental energy vehicle technology would positively impact the carbon emissions reduction^12^. Most of the research compares the static cost assessment and carbon emissions of environmental energy vehicles and traditional energy vehicles. This draws the advantages and disadvantages of different energy type vehicle results. Existing studies are still lacking in considering the impact of dynamic transportation on vehicle costs. Few studies have taken corporate cash flow as a factor in vehicle combination decisions. However, the health of a company’s cash flow is often a necessary condition for determining whether the company can operate sustainably.

In the research of route optimisation, Li et al. proposed the optimisation goal of minimising vehicle energy consumption, carbon emissions, and the total cost of renting vehicles. It established a vehicle routing problem model that considers energy consumption and carbon emissions. The designed tabu search algorithm proves the effectiveness of the model^13^. Duan et al. used vehicle fuel consumption as a carbon emission coefficient, established a heterogeneous vehicle route model with carbon emissions, and determining that appropriatly different types of vehicles can reduce total costs and carbon emissions^14^. Deng et al. proposed the optimal electric vehicle route model for fast charging and regular charging according to the time-of-use electricity price and designed a learnable parthenogenetic algorithm to reduce logistics operation costs^15^. Li et al. used an iterative local search algorithm to solve parcel locker allocation on the planned transportation route. Through case data testing, they believe that this method can effectively reduce carbon emissions in the last-mile delivery^16^. Li et al. proposed the problem of dense semisoft time windows with heterogeneous vehicles in single parking lots. The goal is to reduce the total delivery cost, and a multichromosome genetic algorithm was used to solve the optimal transportation path under transportation cost and penalty cost^17^. Current research focuses on optimisation problems such as minimising transportation costs and minimsing carbon emissions under the determined number of vehicles. However, achieving minimising carbon emissions requires companies to sacrifice more resources to achieve, which is not fully in line with the company's lean management goal: to create the most generous benefits with the least cost. In addition, although the heterogeneous fleets are implemented to reduce transportation carbon emissions in the existing route optimisation model research, most of the research focused on optimising the transportation cost of heterogeneous fleets. It did not comprehensively optimise the purchase cost of environmental energy vehicles as a parameter that affects operating costs. Current research, however, does not fully consider whether the different numbers of heterogeneous vehicles would have different effects on carbon emissions. Moreover, the existing studies take environmental vehicle decisions and vehicle routing problems as independent questions to study.Therefore, it is extremely meaningful for companies to research the integrated decision of multi-energy type vehicles (MTV) combined strategy and transportation route optimisation by considering the sustainable development of companies is meaningful.

In summary, we proposed the integrated decision-making of MTV combined strategy and route optimisation based on the customer demand (IDMTVCROCD) model. Based on the optimisation results of the improved genetic algorithm, we analysed the impact on operating cost and carbon emissions under different MTV combination scenarios. The purpose is to find the most suitable combination of MTVs while ensuring that the transportation plan is met. Furthermore, optimising the transportation route with the combination of MTVs to improves transportation capacity and optimises operating costs and reduces carbon emissions. This is the goal of lean management for optimal resource planning of chemical logistics companies. The problem discussed in this paper is a vital part of the lean management of logistics companies, and also an essential part of improving the differentiated competitiveness and low-carbon transformation of chemical logistics companies. Optimal allocation of transportation equipment resources will improve the chemical logistics companies’ operational resilience and the ability to resist risks.

### Problem description

The essence of the IDMTVCROCD problem is a combinatorial optimisation problem with limited resources. When chemical logistics companies are optimising their operating resources, considering vehicles purchased from the perspective of carbon emissions only, while ignoring the cost-profit margin, companies will prefer to buy as many environmental energy vehicles as possible, or even replace all traditional energy vehicles. However, the sudden increase in fixed costs will inevitably reduces the company’s ability to resist risks and even break the capital chain^18^. In addition, if the MTV combined strategy does not integrate customers’ actual demand, and ignores the cost of carbon emissions of vehicles with loading capacity, carbon emission deductions fail. Therefore, decision making for,chemical logistics companies should be based on customer demand and formulate a reasonable environmental energy vehicle purchase plan under the companies’ optimal operating cost. This decision produces two subproblems: one is the MTV combined strategy under the optimal operating cost; the other is the transportation route planning under the optimal carbon emission.

1. The problem of the MTV combined strategy under the optimal operating cost.

When considering the purchase of environmental energy vehicles by chemical logistics companies, two extreme situations should be avoided. The first extreme situation is to scrap all existing traditional energy vehicles and purchase environmental energy vehicles for transportation. Under this situation, logistics companies face a sudden increase in purchase costs, which causes the companies’ cash flow to be insufficient to support long-term operations, and weaken the company’s ability to resist risks^19^. The second extreme situation is a lack of consideration when purchasing environmental vehicles and keeping traditional energy vehicles for transportation. Under this situation, although companies do not need to invest in environmental vehicles, it causes additional carbon emission costs due to excessive carbon emissions. High carbon emissions also cause a decrease in the company’s reputation within our society where there is an increase in environmental awareness increases. Brand reputation is also an essential part of a company profits.

Therefore, chemical logistics companies should develop a strategy of combined transportation of MTV for optimal operating costs. Meanwhile, companies could achieve operations of high-resilience operation and reduce carbon emissions through the optimal ratio of scrapping existing traditional energy vehicles and purchasing environmental energy vehicles.

(2) Transportation route planning under the optimal carbon emissions.

In optimising logistics transportation businesses, both the minimisation of transportation costs and the carbon emissions of the entire transportation network must be considered. A calculation is carried out based on the type and number of energy sources of vehicles, the freight volume and transportation distance under customer demand, which makes the results more reasonable. The purpose of this paper is to provide a chemical logistic company’s customers with warehousing and transportation services. Multienergy type fleets load goods on trucks from the chemical logistic company's distribution area (0) and are transported from the logistic company to various demand nodes (1–20) (see Supplementary Fig. 1).

When planning transportation routes, for the purpose of environmental protection, each carbon emission company receives a certain amount of carbon allowance every year by following the carbon allowance policy. After the allocated carbon allowance is exceeded, the company needs to buy carbon allowances in the carbon trading market. The additional cost of carbon emissions becomes the operating cost of the company^20^. If the annual carbon allowance is not used up, the company could put the remaining carbon allowance into the carbon trading market for sale to obtain profit. Better known as the carbon allowance difference^21^. Therefore, it is necessary to consider the ideal distribution toute when the carbon allowance difference is optimal when optimising the transporation network.

In summary, according to the above two subproblems, this paper expects the intergrated decision-making model of the MTV combined strategy and route optimisation based on customer demand (IDMTVCROCD) to solve logistics companies, and to use a reasonable number of MTVs for combined transportation. Achieving the optimal operating cost and carbon emissions, this model also considers the impact of transportation routes on operating costs and carbon emissions (see Supplementary Fig. 2).

## Results

This paper uses the Python language to call the Cplex1 2.7 engine for programming and implements an improved genetic algorithm in a computer configuration environment configured with Intel Core i53235m 2.6 GHz CPU 4 GB RAM. For large-scale road networks and complex application scenarios, the decision-making process depends on heuristic algorithms to obtain satisfactory solutions^22^.

### Parameter source of the calculation

The research question of this paper comes from the actual operation of the second largest chemical logistics company with an operating income of 3 billion yuan in China. The data are based on the company;s actual situation, with the collection and sorting are from the actual investigation of the company. This is a comprehensive chemical logistics company that integrates warehousing and transportation functions. Due to the increase in environmental protection awareness and the favour of environmental transportation by the customers it serves, the company’s proactive low-carbon behaviour has been triggered^4^. In the current operation, the company’s hanging professional hazardous chemical vehicles are all diesel vehicles, which emit large amounts of carbon dioxide. This is one of the culprits leading to the greenhouse effect^23^. Therefore, the company urgently needs to configure transportation equipment to rationally carbon emissions rationally. It also aims to company brand reputation in the fierce chemical logistics market and to implement a differentiated competition strategy to ultimately achieve lean logistics management for the company. Based on this, we establish customer demand-oriented MTV combined with transportation and route optimisation integrated decision-making for chemical logistics companies. We expect this integration to be a more reasonable achievement for companies’ initiatives to save energy and reduce emissions.

In the case analysis, a distribution plan is formulated based on existing customer orders. The case selects 20 customer nodes for analysis. Each customer’s demand is different (see Supplementary Table 1).

Chemical logistics is different from ordinary logistics businesses; it is unique in both warehousing and transportation links. Specialised hazardous chemical storage tanks and containers should be used for transportation; towing tractors should be used for transportation. Vehicles need to be cleaned and disinfected every time they return to the logistics company after serving customers to prevent residual hazardous chemicals from causing potential risks^24^. Therefore, when the company formulates integrated decision-making of vehicle combined strategy and route optimisation based on customer demand, the company needs to consider customer demand and proper vehicle scheduling.

**Figure 1:**
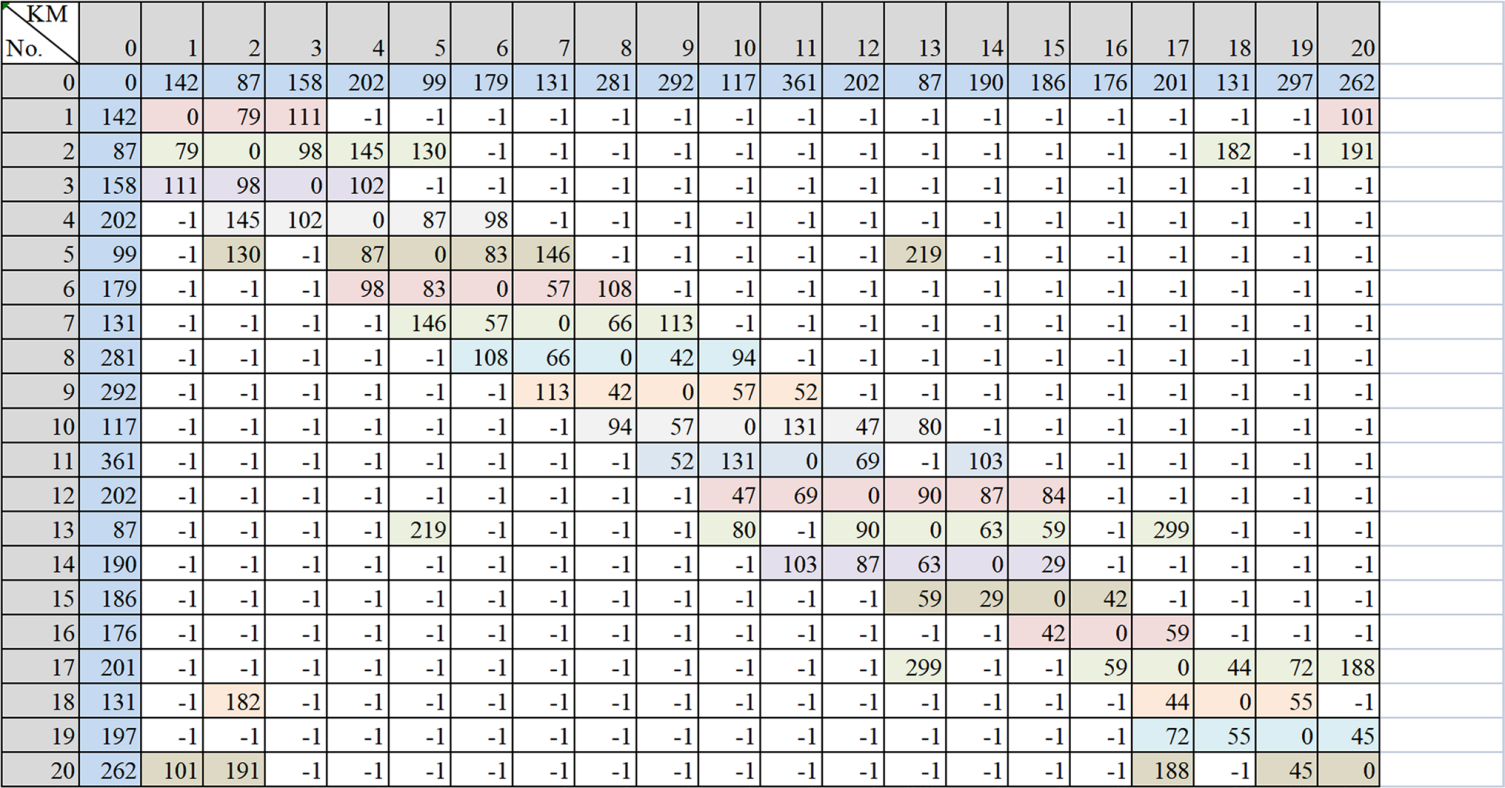
Transportation distance information from chemical logistics company to customer nodes. If the distance between customers is displayed as − 1, the road is disconnected. If the numbers are displayed, it represents the transportation distance.

As the transportation demand network defined in Supplementary Fig. 1, the transportation network between all customers is not all connected due to the limited loading capacity of vehicles and geographical constraints. Figure 1 shows the transportation distance information of the distribution area and customer nodes.

When the company makes an integrated decision, based on the current customer demand to upgrade transportation equipment, it first needs to consider the various purchase costs and subsidy policies. In addition, calculation of the various expenses that will be incurred is necessary. According to the definition of using the present value calculation in this paper, all cost calculations use the present value method. The cost parameters are as follows.

1. According to the truck trading website data, the new energy (electric) tractor model is selected. The company chooses to purchase the environmental vehicle model as the ‘M3000 electric tractor’ with a loading capacity of 39 tons. Therefore, the present value of the environmental vehicle purchase cost (excluding subsidies) $C_{ev}$ is 728,500 yuan.
2. The maintenance cost of environmental vehicles mainly comes from annual routine maintenance. Daily maintenance includes tire replacement, brake pad replacement, and replacement parts used by wear and insurance. Therefore, the present value of the maintenance cost of environmental energy vehicles $C_{me}$ is 127,400 yuan.
3. With the continuous innovation of environmental vehicle technology, the life of the battery packs is gradually increasing. However, due to the vehicle frequency charging times and the high annual mileage, the battery needs to be replaced when it decays to 70%. Therefore, the battery replacement cycle is every five years. Furthermore, the present value of the battery replacement cost $C_{b}$ after five years is 74,200 yuan.
4. The government encourages carbon emission companies to reduce their carbon emissions; appropriate vehicle purchase subsidies have played a particular role in encouraging logistics companies to purchase environmental vehicles^24^. According to the current subsidy policy for purchasing environmental vehicles in Shandong Province, the present value of the subsidy for purchasing environmental vehicles $C_{s}$ is 50,000 yuan.
5. The company expects to purchase an environmental vehicle with a driving range of 200 km and a peak power of 350 kW. According to the charging standard of the environmental vehicle charging pile in Shandong Province, it is 0.9 yuan per kilowatt. The present value of the electrical cost of the environmental vehicle $C_{p}$ is 1.39 yuan/ton kilometre.
6. The maintenance content of traditional vehicles is the same as that of environmental vehicles, with the present value of maintenance cost $C_{mo}$ being 155,400 yuan.
7. The present value of subsidies for early scrapping of traditional vehicles $C_{a}$ is 50,000 yuan.
8. Currently, the traditional vehicles used by the company are fully loaded with 38.5 tons. The current diesel price in Shandong Province is 5.19 yuan/L, resulting in a present value of fuel cost of traditional vehicles of 1.87 yuan/ton-kilometre.
9. According to the technical guidelines for coordinated control of urban traffic air pollution and greenhouse gases, the current cost of environmental pollution caused by carbon dioxide emissions $C_{e}$ is 1.42 yuan/kg, and the value of $T_{x}$ is 1.08. According to the international carbon emission calculation formula and coefficient from the ‘China Emissions Trading’ Website, $\lambda$ is 2.778 kgco_2_/L. Therefore, the calculation method of the carbon emission cost $C_{e} \cdot \lambda D_{ij} \times \left( {a \cdot z_{ik} + b} \right) \cdot x_{ijk}$. The carbon emission cost can be obtained as 1.42 × 2.778 × $D_{ij} \times \left( {a \cdot z_{ik} + b} \right) \cdot x_{ijk}$, where *a* = 3.650*10^−3^ and *b* = 0.2125.

### The integrated decision-making scheme of vehicle combined strategy and route optimisation based on customer demand

**Figure 2:**
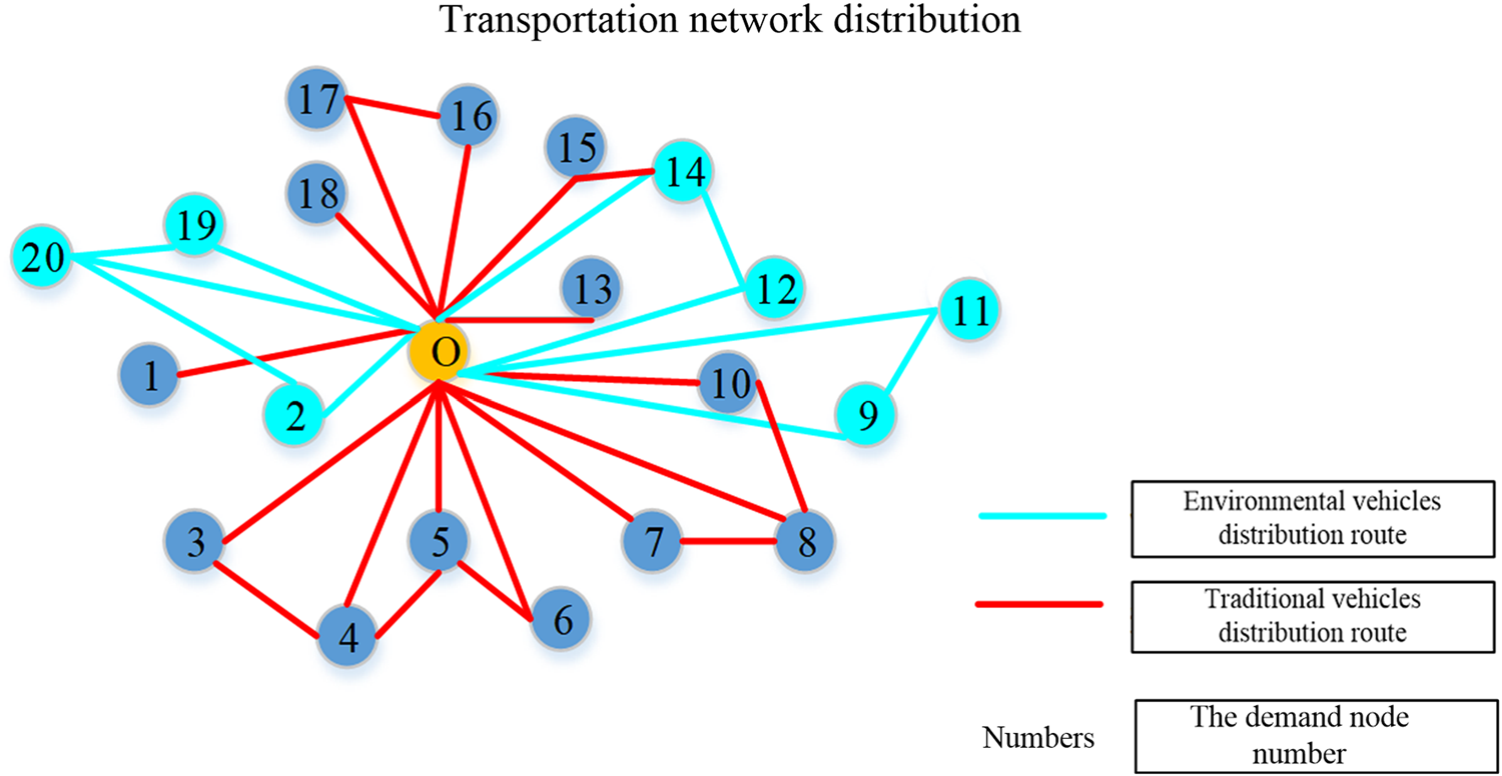
Route optimisation scheme for MTV considering the satisfaction of customer demand.

The model solution based on the discussed steps is designed by an improved genetic algorithm. According to this algorithm, the route optimisation scheme of MTV is obtained. The optimisation scheme in Fig. 2 shows that the logistics company's vehicle purchase strategy is to scrap part of their traditional vehicles and purchase a certain number of environmental vehicles to form an MTV combined fleet for transportation. The optimisation scheme not only meets the sustainable operation purpose of the company but also reduces carbon emissions and finally achieves lean logistics management.

Because the scheme involves customer demand, company cash flow surplus, cost of existing transport vehicles and the purchase of transport vehicles, the transportation route is limited by the transportation of hazardous chemicals. It is a comprehensive and complex integrated decision, and the final solution is more practical.

According to the optimisation results obtained after the computer solution, the 14 traditional vehicles of the company are selected as case samples. 4 traditional vehicles should be scrapped, and 4 environmental vehicles should be purchased to form an integrated decision-making plan for vehicle combined strategy and route optimisation based on customer demand. Table 1 is the MTV routing optimisation results.

**Table 1:** MTV routing optimisation results.

No	Vehicle model	Route	Demand (Ton)	Full load rate (%)
1	Environmental vehicle	0–19–20–0	39	100
2	Environmental vehicle	0–20–2–0	39	100
3	Environmental vehicle	0–11–9–0	39	100
4	Environmental vehicle	0–12–14–0	39	100
5	Traditional vehicle	0–1–0	27.2	70.64
6	Traditional vehicle	0–15–14–0	38.5	100
7	Traditional vehicle	0–3–4–0	38.5	100
8	Traditional vehicle	0–5–4–0	38.5	100
9	Traditional vehicle	0–5–6–0	38.5	100
10	Traditional vehicle	0–18–0	26.6	69.09
11	Traditional vehicle	0–7–8–0	38.5	100
12	Traditional vehicle	0–10–8–0	38.5	100
13	Traditional vehicle	0–13–0	32.2	83.63
14	Traditional vehicle	0–16–17–0	38.5	100

The optimisation results indiciate that when the company makes decisions on the reorganisation of transportation resources under the influence of proactive low-carbon behaviour, the optimisation results of the MTV combination meet customer demand to the greatest extent. The results also show that if a company purchases environmental vehicles based on optimal operating costs, the goal of reducing carbon emissions can also be achieved. Therefore, the integrated decision-making of vehicle combined strategy and route optimisation based on customer demand in this paper has strong advantages.

### Algorithm convergence analysis

**Figure 3:**
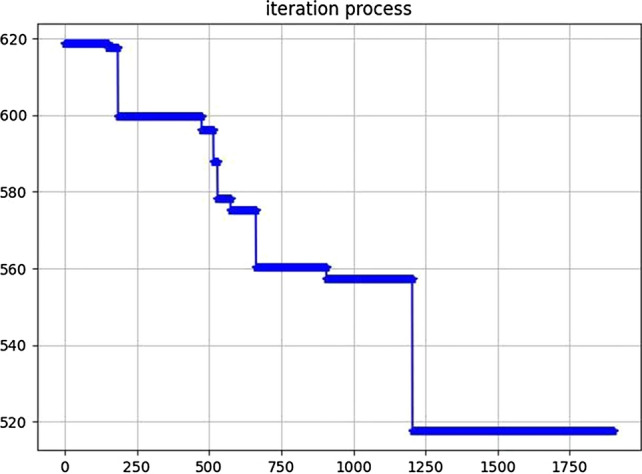
Algorithm convergence graph.

To test the computational efficiency and convergence performance of genetic algorithms, the dynamic curve of the objective function value of the algorithm with the number of iterations is drawn, as shown in Fig. 3.

We obtained that the convergence of the fitness value becomes stable after approximately 1240 iterations from the convergence graph result. Through the observation of the objective function, we concluded that there are fluctuations in the process of convergence, but the entire evolution direction is guaranteed. The improved genetic algorithm has a faster convergence speed than the traditional genetic algorithm. Moreover, the algorithm can better obtain the optimal value of operating costs and carbon emissions, avoiding the premature problem of genetic algorithm, which effectively prevents the algorithm from falling into the optimal local solution. This quickly eliminates individuals with low fitness, finding the optimal solution more efficiently. When solving the IDMTVCROCD problem, its convergence speed has more advantages and a more robust global searchability. In summary, the improved genetic algorithm proposed in this paper has strong feasibility and effectiveness.

### Cost analysis under the different quantities of environmental vehicle procurement strategy

**Figure 4:**
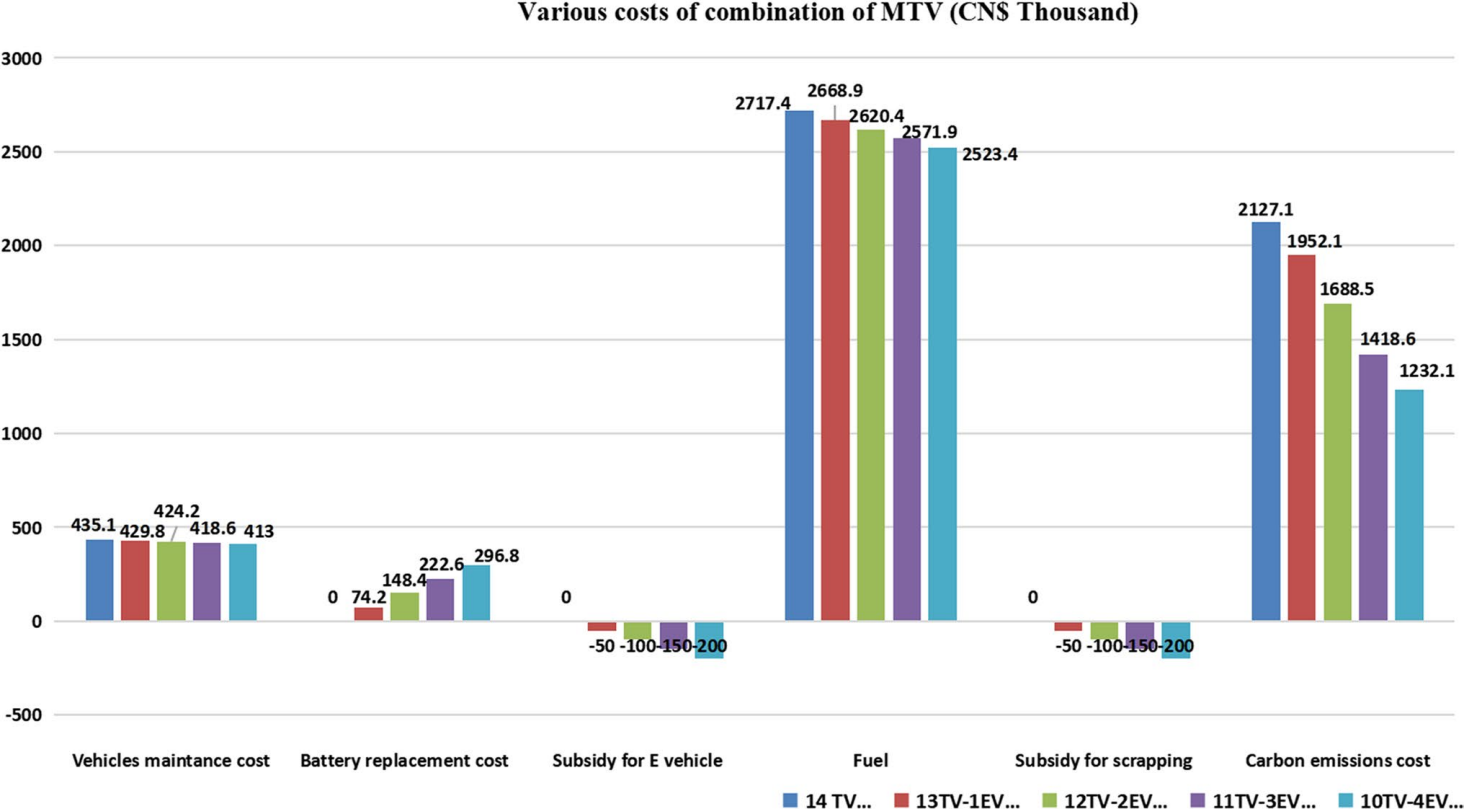
Comparison of various costs of different combinations of MTV.

**Figure 5:**
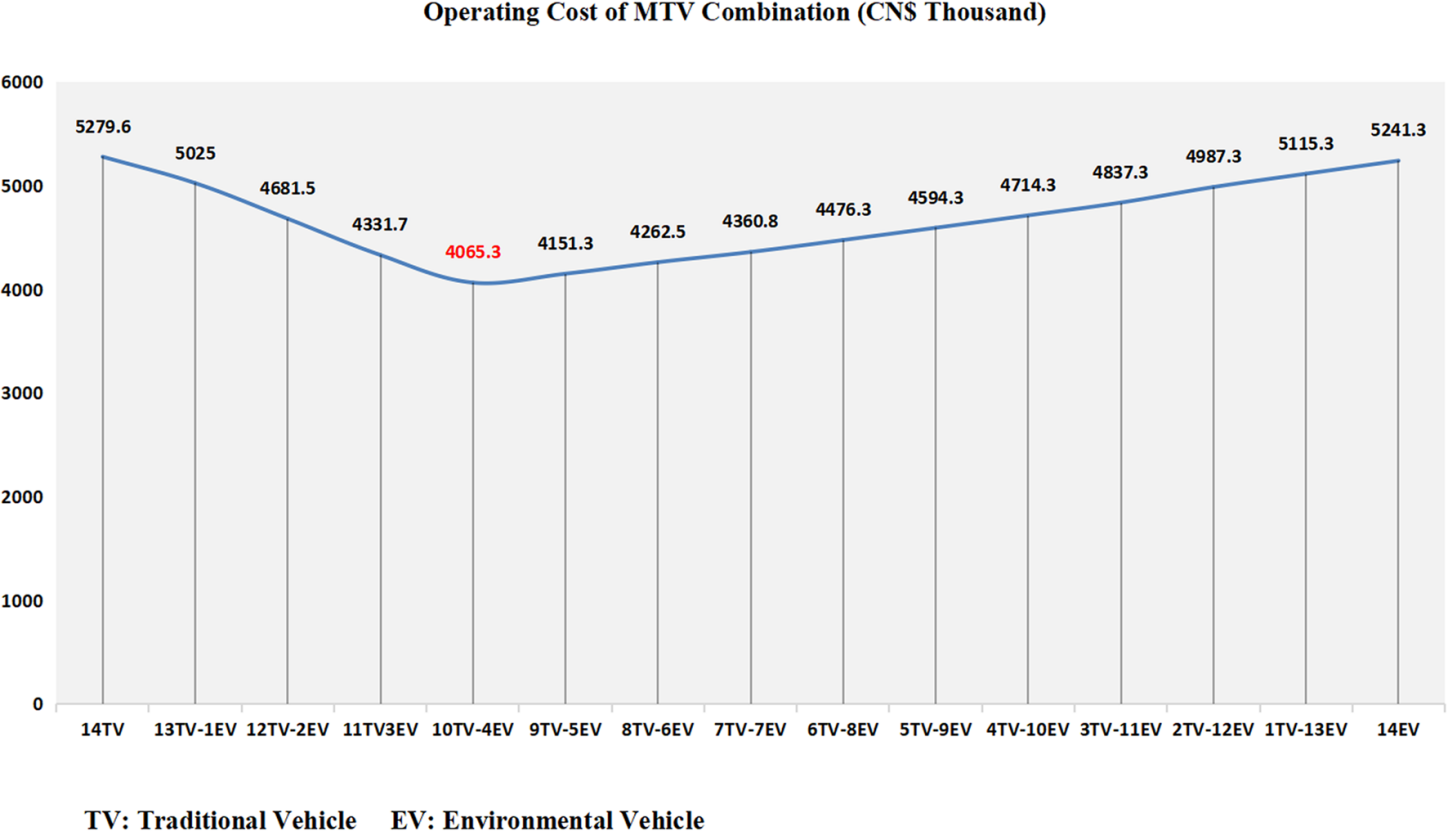
Operating costs of different numbers of MTV combinations.

(1) To further compare the impact of different MTV numbers on operating cost, we compare the costs of five groups of different MTV combinations based on the same customer demand. According to the data in Fig. 4, under the same scale of customer demand, the vehicles’ maintenance cost and fuel cost do not show significant changes with the different combinations of environmental vehicles (EVs) and traditional vehicles (TVs). Moreover, according to the optimised transportation route based on customer demand and the carbon emissions calculation model, the carbon emission cost of the loading vehicles is accurately calculated. By comparing the cost of carbon emission during the combined transportation of different numbers of MTVs, we find that purchasing a certain number of environmental vehicles for transportation significantly reduces vehicles’ total carbon emission cost. Figure 4 shows that environmental vehicles have played a positive role in reducing the carbon emissions of the logistics company. It also shows that the integrated decision-making scheme of the vehicle combined strategy and route optimisation proposed in this paper has achieved the goal of reducing carbon emissions. The reduction in the cost of carbon emissions has also achieved the goal of energy savings and emission reduction in lean logistics management.

(2) To further understand the problem, we can analyse the operating costs of different combinations of MTV and discuss the differences the company has. Figure 5 shows that the combination of different numbers of traditional transport vehicles (TVs) and environmental transport vehicles (EVs) combined with transportation route planning has resulted in changes in operating costs.

When the company develops a vehicle upgrade plan, choosing to replace a select number of traditional vehicles with environmental vehicles gradually reduces its operating cost. This is mainly because environmental vehicles do not produce carbon emissions during transportation, and the cost of carbon emissions is a part of the operating cost. However, with the gradual increase in the number of environmental vehicles, after the critical point of combined transportation of 10 traditional vehicles and 4 environmental vehicles, the addition of one environmental vehicle and scrapping one traditional vehicle will increase the company’s operating cost. Moreover, through the analysis of operating costs, we find that the logistics company has almost the same operating cost for using all traditional vehicles or environmental vehicles for transportation at the current stage. In summary, although carbon emissions are inversely proportional to the use of environmental vehicles, a company’s expenditure can become too high, due to unreasonable purchase plans of environmental vehicles. Under the current customer demand scale, it eventually leads to an increase in operating costs, which contradicts the purpose of reducing costs and increasing efficiency in lean logistics management.

(3) Limited by the current technology of environmental vehicles and the high mileage of electric tractors each year, the battery needs to be replaced with five year period. Short period battery replacement has also become one of the limiting factors for the logistics companies in formulating vehicle upgrade strategies^25^. According to the experimental results of scientists in China, Germany, and France, lithium-ion battery technology has achieved a life span of 16 years in a laboratory environment with a cruising range of 2 million km^26^. It can be inferred that with the development of technology in the future, the heart of battery cost analysis for environmental vehicles under the scenario that a reduced battery cost of 50% along with the service life being double.

In the future, as an alternative to lithium-ion batteries, sodium-ion batteries will be more widely used. From the perspective of battery manufacturing, the reason that the global reserves of sodium resources are abundant, and the distribution is uniform, is that the mining cost can be greatly reduced. In addition, the anode and cathode current collectors of sodium-ion batteries can use lower-cost aluminum foil^27^. From a battery life perspective, the current high and low temperature tests of sodium-ion batteries show that their performance is better than that of lithium-ion batteries. In addition, because the internal resistance of sodium-ion batteries is slightly higher than lithium-ion batteries, the current experimental environment shows less instantaneous heat generation, leading to a safer performance^28^. Therefore, when battery manufacturing technology becomes more mature, the cost of batteries used in electric vehicles will drop significantly.

**Figure 6:**
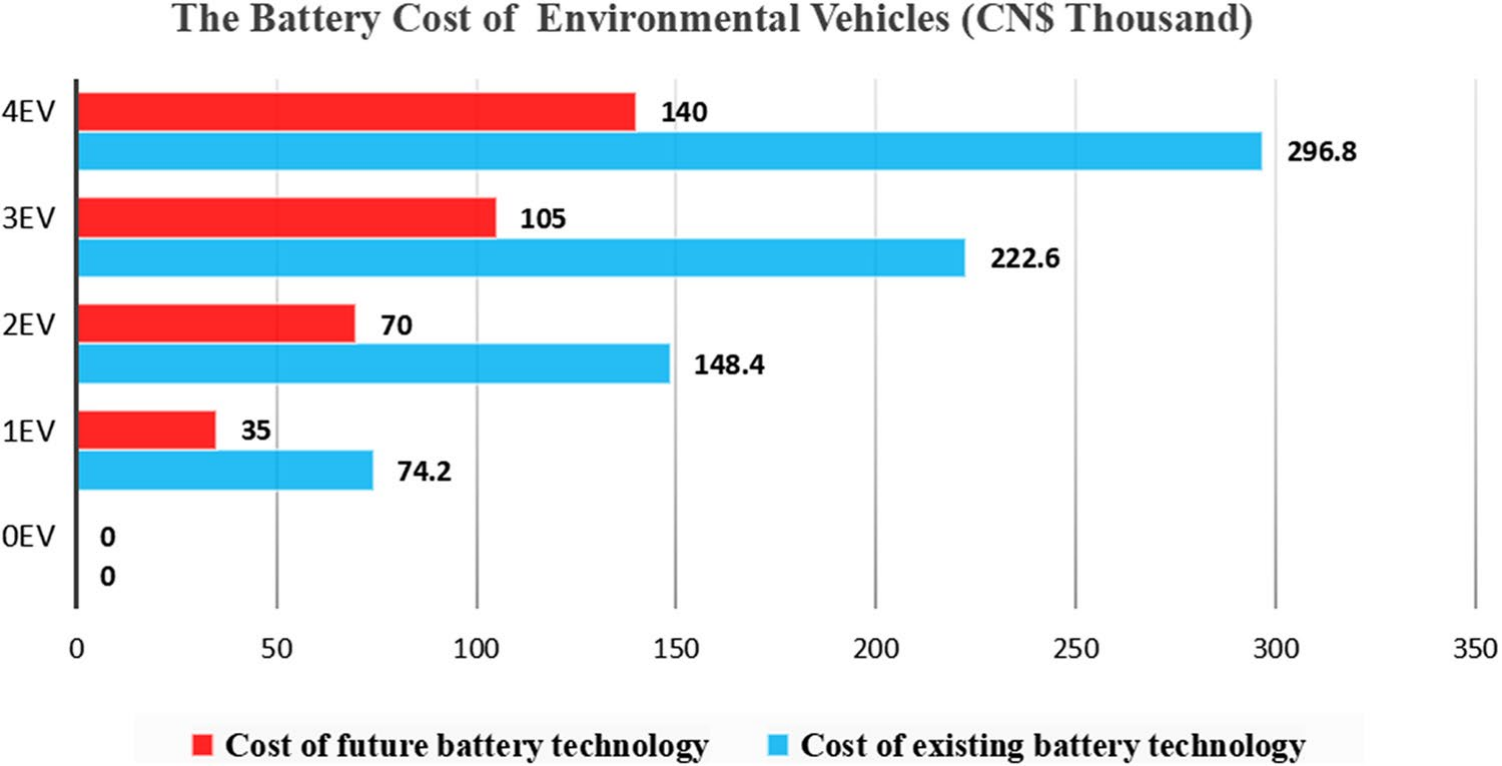
The battery cost analysis of environmental vehicles. Cost comparison of two different battery technologies.

According to the battery cost in different battery scenarios in Fig. 6, it can be concluded that if the cost of the battery is reduced by 50% in the future and the service life is doubled, the cost of changing batteries of environmental vehicles (EVs) will be significantly reduced. The related operating cost will also decrease, and the company's cash flow will increase accordingly. At that time, the company will be more biased towards environmental vehicles when making vehicle purchase decisions.

**Figure 7:**
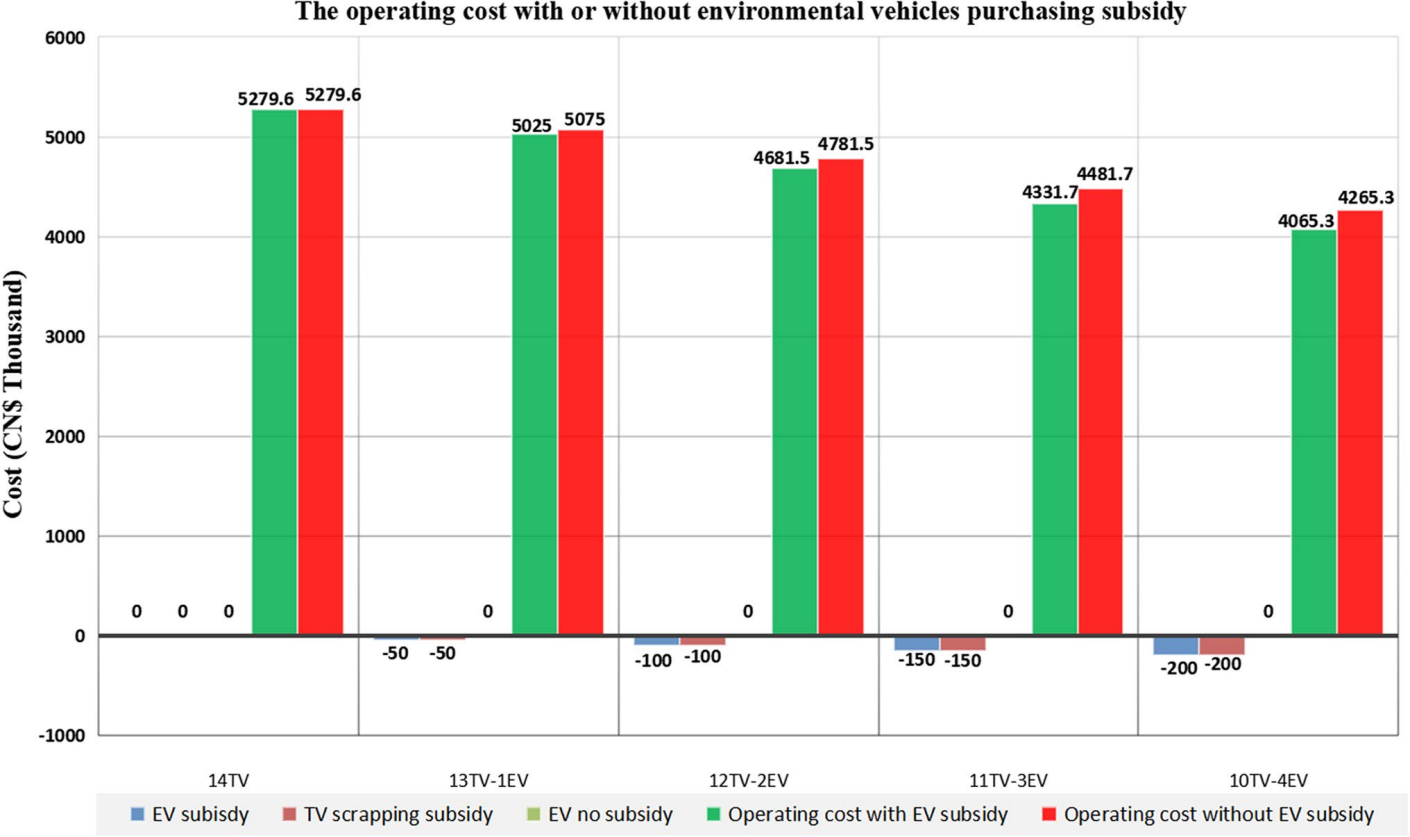
Changes in subsidy for the purchase of environmental vehicles. The results in this figure mainly compare the operating cost with or without environmental vehicles purchasing subsidy.

(4) At present, the environmental vehicle manufacturing technology is becoming increasingly mature. With the continuous innovation of technology and the continuous reduction of costs, environmental vehicle industries are also undergoing structural changes. Therefore, with a maturing industrial structure, the manufacturing cost of environmental vehicles will continue to decline, and the market share will continue to increase. The subsidy policy for purchasing environmental vehicles will gradually withdraw from the market compensation mechanism. In this paper, we pay attention to the impact of the company's proactive low-carbon behaviour on purchase strategy. With the gradual withdrawal of subsidy policies, we analyse the impact on the company decision-making when environmental vehicles do not purchase subsidies. Figure 7 below shows the changes in subsidies for the purchasing of environmental vehicles. Shown with the gradual withdrawal of subsidy policies, we analyse the impacts on the company’s decision-making without the purchase subsidy.

Figure 7 shows that if the number of environmental vehicles purchased by the company is small, the cancellation of the subsidy policy does not bring apparent changes to the company's operating cost. However, with the gradual increase in the number of environmental vehicles purchased by the company, the trend of the operation cost of the company has also shows a gradual increase after the withdrawal of the subsidy policy. The figure shows that if there is subsidy policy withdrawal, the more environmental vehicles purchased, the more costs the company has to pay. Therefore, the vehicle purchase subsidies for environmental vehicles have played a positive role in developing vehicle purchase strategies for companies. Only when the current state of manufacturing technology is more mature for environmental vehicles can the manufacturing cost be lower than now. Furthermore, the sales price of environmental vehicles can be reduced to offset the subsidy's benefits. At that time, the withdrawal of the subsidy policy will no longer affect the company's decision.

## Discussion

Energysavings and emission reduction are important goals of lean logistics management, and it is also the consensus of society and humankind for environmental protection is reached. In this paper, in the proactive low-carbon behaviour of a chemical logistics company, based on the customer demand, we comprehensively consider the mechanism of MTV combined transport and route optimisation on the company operating cost and carbon emissions. We developed an integrated decision based on customer demand for MTV combined strategy and route optimisation. Intergrated decision-making analyses the various costs of combined transportation on a varity of different environmental vehicles and existing traditional vehicles based on customer demand. In addition, we are optimising the transportation route under the MTV combined strategy along with introducing the carbon emission calculation function under the vehicle's transportation route with freight. The combined transportation of MTVs reduces the risk of upgrading transportation equipment for the chemical logistics companies.

The ultimate goal of an independent environmental vehicle purchase plan or the vehicle route optimisation plan is to provide energy savings, emission reduction, cost reduction and efficiency enhancement. When combining the two plans, an integrated decision-making plan has a more comprehensive and practical consideration of operating cost and carbon emissions.

The problem studied in this paper is complex joint optimisation. According to the characteristics of the model, an improved genetic algorithm is designed to solve this problem. The optimal solution under the trade-off between operating costs and carbon emissions is obtained and the effectiveness of the combined transportation strategy of MTVs in reducing operating costs and carbon emissions is proven. Finally, a numerical analysis is carried out based on the field survey data of the chemical logistics company. The case indicates that (1) an enhanced genetic algorithm can improve the algorithm's global convergence ability and result in accuracy, (2) the optimised combination of environmental vehicles and traditional vehicles have played a positive role in reducing carbon emissions, and (3) although environmental energy vehicles have a positive effect on reducing carbon emissions, unreasonable upgrading of vehicles will cause excessive operating costs for the chemical logistics company. The chemical logistics company should make a rational combination of vehicles based on the goal of optimal operating cost to enhance business resilience, (4) the cost reduction of environmental vehicles has a positive impact on decision-makers to formulate the MTV strategy. Future studies can consider the congestion problem of transportation routes, including the impact of congestion costs on operating costs.

## Methods

An improved genetic algorithm is designed to solve the IDMTVCROCD model. This section is based on optimisation theory and uses the dynamic programming algorithm of operations research to solve the problem.

### Model assumption

1. Service site assumption.
  Chemical logistics companies have only one cargo distribution area, which serves as the starting point for transport vehicles and a return destination after delivery.
2. Vehicles assumption.
  Chemical logistics companies have two types of energy transport vehicles: traditional vehicles (diesel energy) and environmental vehicles (electric energy). The number of vehicles is limited, and the vehicles are required to turn off their engines when serving customers.
3. Loading assumptions.
  The loading capacity of transport vehicles does not exceed the vehicle's maximum loading limit, and the total customer delivery demand for each vehicle does not exceed the vehicle's load.
4. Demand assumption.
  The number of customers to be served and the service area are known, and each customer can accept no more than three transport vehicles for service to meet demand.

### Symbols and parameter description

The symbols and meanings used in model construction are shown in the supplementary Table 2.

### Model establishment

The mathematical description of each objective function is introduced as follows.

1. Proactive low-carbon behaviour of chemical logistics companies.

The proactive low-carbon behaviour of logistics companies is the positive correlation coefficient of the demand for environmental transportation tools, which can also be expressed as $T_{x} = T_{1} \left( x \right) + \pi a_{x}$, where $T_{1} \left( x \right)$ is the demand for transportation tools when the logistics companies do not consider carbon emissions, and $a_{x}$ is the environmental protection degree of the transportation tools.

(2) The cost of environmental vehicles.

When considering the cost, the present value could more accurately reflect the time value of money and take financial risks and other considering factors^29^. $I$ is used to indicate the discount rate. According to the current central bank’s loan interest rate for the same period as a guide, the discount rate is 4.35%. Use $n$ to indicate the number of periods, usually in years. Use $L$ to indicate the vehicle’s service life. The costs of environmental vehicles are as follows:

1. The purchase cost: $C_{ev} = \sum\nolimits_{n = 0}^{L} {\frac{{C_{evn} }}{{(1 + I)^{n} }}}$.
2. The maintenance cost: $C_{me} = \sum\nolimits_{n = 0}^{L} {\frac{{C_{men} }}{{(1 + I)^{n} }}}$.
3. The cost of battery: $C_{b} = \sum\nolimits_{n = 0}^{L} {\frac{{C_{bn} }}{{(1 + I)^{n} }}}$.
4. Subsidies for the purchase of environmental vehicles: $C_{s} = \sum\nolimits_{n = 0}^{L} {\frac{{C_{sn} }}{{(1 + I)^{n} }}}$.
5. The fuel cost of environmental vehicles, which are mainly lithium iron phosphate batteries: $C_{p} = \sum\nolimits_{n = 0}^{L} {\frac{{C_{pn} }}{{(1 + I)^{n} }}}$.

(3) The cost of traditional energy vehicles. Using the cost calculation method of traditional energy vehicles, the present value method is still to be used for calculation.

1. The maintenance cost of traditional vehicles: $C_{mo} = \sum\nolimits_{n = 0}^{L} {\frac{{C_{mon} }}{{(1 + I)^{n} }}}$.
2. Subsidies for scrapping existing traditional vehicles. According to government policy, early scrapping of high carbon emission diesel vehicles is given corresponding subsidies: $C_{a} = \sum\nolimits_{n = 0}^{L} {\frac{{C_{an} }}{{(1 + I)^{n} }}}$.
3. The fuel cost is based on diesel. The fuel cost of traditional vehicles: $C_{f} = \sum\nolimits_{n = 0}^{L} {\frac{{C_{fn} }}{{(1 + I)^{n} }}}$
4. When calculating the carbon emission cost of traditional vehicles, the first step is to calculate the carbon dioxide emissions of traditional vehicles. The cost of environmental pollution is multiplied by the amount of carbon dioxide emissions to obtain the cost of carbon emissions, which is $C_{e} \cdot CF$. For road transport vehicles, the carbon emission data should be obtained by multiplying the data in the vehicle service process with the carbon emission factor to obtain the carbon emission data of the vehicle during the service process^30^. In the data collection process, we focus on the data generated in the service process of vehicles. That is, fuel, cargo load, transportation distance, and vehicle driving conditions. We use the improved MEET (Methodologies for estimating air pollutant emissions from transport) method to calculate the carbon emissions^31^. The direct carbon emissions $CF$ of vehicles should be equal to the product of fuel consumption $F_{c}$ and fuel conversion factor $\lambda$, namely
  1 $$ CF = F_{c} \times \lambda $$

When calculating the fuel consumption of traditional vehicles, carbon emissions depends on fuel consumption, and fuel consumption depends on transportation distance and has a positively linear relationship to the load. Taking the driving distance $D_{ij}$ of the transport vehicle to the customer, the weight load $z_{ik}$ of the transport vehicle, $k$ carrying the goods required by the customer, $i$, and the possible gradient factor $G$ in the road as input, calculate the fuel consumption function of traditional vehicles in the process of transporting goods as:

2 $$ F_{c} = D_{ij} \times (z_{ik} \times a + b) \times G $$

where $a$ represents the fuel consumption factor and $b$ represents the driving resistance factor. Fuel consumption parameters are related to the model, emissions, and cargo volume of traditional vehicles. The driving resistance coefficient depends on the load rate of the engine of the traditional vehicles. Then, we assume that when a vehicle serves customers on the road with a slope of 0, the engine movement of a traditional energy vehicle will produce carbon emissions by its self-load rate. Therefore, the Eq. (2) can be converted to

3 $$ F_{c} = D_{ij} \times (z_{ik} \times a + b) \times x_{ijk} $$

Finally, the calculation method for carbon emissions of the traditional vehicles is obtained by

4 $$ \lambda D_{ij} \times (a \cdot z_{ik} + b) \cdot x_{ijk} $$

(4) Carbon allowance differencez

Carbon allowances refer to the issuance to various industries by the carbon regulatory authorities, expressed as $CE_{q}$, and the total carbon emissions generated by the logistics company are expressed as $CE$. We could obtained $CE - CE_{q} = CE_{dv}$, where $CE_{dv}$ is the carbon allowance difference. When $CE_{dv}$ < 0, it means that the company has a surplus in the total carbon allowance issued through energy-saving and emission reduction measures. The company can sell the surplus carbon allowance to the carbon trading market where economic benefits can be obtained. Therefore, $CE_{dv}$ < 0 indicates that the company is in the best state of carbon emissions^32^.

In summary, with the optimal operating cost and optimal carbon emissions as joint optimisation goals, an integrated decision model for the MTV combined strategy and route optimisation based on customer demand is established as follows:

5 $$ C = \min \frac{{f_{1} - f_{2} + f_{3} + f_{4} + f_{5} }}{{T_{x} }} $$

where

5.1 $$ f_{1} = (C_{ev} + C_{me} + C_{b} - C_{s} ) \cdot N $$

5.2 $$ f_{2} = C_{a} \cdot (E - M) $$

5.3 $$ f_{3} = C_{mo} \cdot M $$

5.4 $$ f_{4} = C_{f} \sum\limits_{k \in M} {\sum\limits_{i \in R} {\sum\limits_{j \in R} {D_{ij} \cdot (a \cdot z_{jk} + b) \cdot x_{ijk} } } } $$

5.5 $$ f_{5} = C_{p} \sum\limits_{k \in N} {\sum\limits_{i \in R} {\sum\limits_{j \in R} {D_{ij} \cdot z_{jk} \cdot y_{ijk} } } } $$

6 $$ CE_{dv} = \min \frac{{C_{e} \lambda \sum\limits_{k \in M} {\sum\limits_{i \in R} {\sum\limits_{j \in R} {D_{ij} (a \cdot z_{jk} + b)} \cdot x_{ijk} } } }}{{T_{x} }} - CE_{q} $$

s.t.

7 $$ x_{ij} + x_{ji} \le 1,\;\forall i,j \in R\backslash \{ 0\} \;{\text{and}}\;i < j $$

8 $$ z_{ij} \le Q_{v} x_{ij} ,\;\forall i,j \in R $$

9 $$ \sum\limits_{i \in R} {x_{ij} } \ge 1,\;\forall i \in R\backslash \{ 0\} $$

10 $$ \sum\limits_{j \in R} {x_{ij} } \ge 1,\;\forall j \in R\backslash \{ 0\} $$

11 $$ \sum\limits_{i \in R} {x_{i0} } \le k $$

12 $$ \sum\limits_{k \in V} {\sum\limits_{{j \in R_{c} }} {x_{0j} } } = 1,\quad \sum\limits_{k \in V} {\sum\limits_{{j \in R_{c} }} {x_{j0} } } = 1 $$

13 $$ \sum\nolimits_{{j \in R\backslash \{ 0\} }} {z_{0j} } = \sum\nolimits_{{j \in R\backslash \{ 0\} }} {D_{j} } $$

14 $$ z_{ij} \ge d_{j} x_{ij} ,\;\forall i \in R,\;\forall j \in R\backslash \{ 0\} $$

15 $$ z_{i0} = 0,\;\forall i = R\backslash \{ 0\} $$

16 $$ x_{ij} \in \{ 0,1\} ,\;\forall i,j \in R $$

The objective function (5) represents the optimal operating cost. The objective function (6) represents the optimal carbon emissions of the logistics company, which is the difference between the total carbon emissions and the carbon allowance allocated to the company so that the allowance difference is optimal.

Constraint (7) is that the route between two customers of the delivery cannot exceed 1. Constraint (8) ensures that the vehicle must be transported on a prescribed transport route during transportation, and the cargo carried shall not exceed the maximum load of the vehicle. Constraints (9) and (10) stipulate that each customer is served by each transport vehicle and served at least once. Constraint (11) limits the number of transport vehicles not to exceed the number of vehicles owned by the logistics company. Constraint (12) means that any transport vehicle is only allowed to leave and return to the distribution area of the logistics company once. Constraint (13) ensures that the total amount of goods loaded on the transport vehicle is equal to the sum of the total amount of goods dispatched to customers. Constraint (14) guarantees that the cargo load when the transport vehicle arrives at any customer is not less than the customer’s demand. Constraint (15) stipulates that transport vehicles returning to the logistics enterprise will no longer deliver goods. Constraint (16) is the integer constraint of the model decision variable 0–1.

### Improved genetic algorithm design

Generally, crossover and mutation are essential links that produce offspring and determine the quality of offspring, thus being the key to the quality of genetic algorithms^33^. The algorithm combines the characteristics of IDMTVCROCD and optimises it in multiple specific operations such as coding structure, crossover and mutation. The optimised algorithm steps are as follows.

S1: *Coding design.* In the combinatorial optimisation problem of vehicles combined and route optimisation, the individual length is expressed as $V \times V \times \left( {M + N} \right)$, where $V$ is the number of nodes, $M$ is the number of fuel vehicles, and $N$ is the number of environmental vehicles. This type of chromosome encoding can effectively distinguish the path schemes of different types of vehicles and ensure that all vehicles can return to the distribution area of the chemical logistics company after serving the demand nodes, forming a subpath. Until all demand nodes are served, a set of feasible solutions is obtained.

S2: *Initialise the population.* According to the constraint conditions, road network structure and customer demand, randomly generate initial solutions that meet the conditions and insert 0 in the first and last genes. This indicates that the vehicle starts from the distribution area and must return to the logistics company distribution area after completing the distribution task. According to the load and transportation conditions, calculate the value of $\sum\nolimits_{i} q$. If it is less than the demand of the task and more than the demand of the next customer, insert 0 between the current customer and the next customer (see Supplementary Fig. 3).

S3: *Fitness evaluation.* When the difference between each individual’s fitness $f(x)$ is not large, it is difficult to use the fitness function to distinguish between individuals, which leads to a similar probability of each individual being selected and causes the selection function of the algorithm to be weakened. This paper uses the classic algorithm of operations research for dynamic programming to improve it. The formula is as follows.

17 $$ f_{(x)} = Z - Z_{\min }^{k} + \xi^{k} $$

$f_{\left( x \right)}$ represents the fitness function, $Z$ represents the objective function value, $z_{\min }^{k}$ represents the minimum objective function value of the current individual, and $\xi^{k}$ represents the adjustment coefficient. As the number of iterations $k$ increases, it gradually decreases. When it is the first-generation individual, it represents infinity. After adding the adjustment coefficient, the gap between excellent and flawed individuals can be increased. The diversity of the population is maintained, and premature maturity is further avoided.

Therefore, there is still a correlation between the fitness function and the objective function of the original problem, but it distinguishes individuals of different generations. According to the research focus, penalising individuals who violate the model’s constraints is appropriate to ensure that the smaller the individual's fitness is, the smaller the probability of being selected. For example, a judgment rule is designed. After the vehicle has serviced node $i$, if its load is 0 and if the next node it serves is not 0, the penalty is increased to ensure that it returns to the distribution area of the chemical logistics company.

S4: *Select.* Calculate and record the fitness value of each individual in the population based on the fitness function. Generate a matrix of individual fitness values sorted by size and adopt the classic roulette selection method.

18 $$ p_{i} = {\raise0.7ex\hbox{${f_{i} }$} \!\mathord{\left/ {\vphantom {{f_{i} } {f_{sum} }}}\right.\kern-\nulldelimiterspace} \!\lower0.7ex\hbox{${f_{sum} }$}} $$

Calculate $p_{i}$ and $f_{sum}$ in the *t*th generation, randomly generate a random number between [0, 1], calculate $s = rand\left( \cdot \right) \times f_{sum}$, calculate the smallest $k$ in $\sum\nolimits_{i = 1}^{k} {f_{i} } \ge s$, and then select the *k*th individual is selected.

S5: *Crossover.* Crossover is an important means for the genetic algorithm to iterate to the optimal solution. It is improved from the crossover mechanism and crossover probability. In terms of the crossover mechanism, due to the unique structure of the vehicles combined strategy and route optimisation problem, the chromosome codes are ordered within groups, these characteristics are of random disorder between the groups.

If the traditional crossover method is used, an across node is randomly generated; however, it is not easy to ensure that the excellent parent gene can continue to the offspring. Moreover, since each parent individual is randomly generated, there may be a problem that the two-parent individuals are the same as the parent after crossing over (see Supplementary Fig. 4). Therefore, combined with the coding method of this problem, a node with chromosome 0 is designed to randomly generate a crossover that retains the existing part of the optimisation plan (see Supplementary Fig. 5).

In selecting the crossover probability, the traditional crossover probability is set as a fixed constant. This constant is not used to propose probabilistics algorithms suitable for different objective functions for specific problems. This paper propose an improved crossover probability calculation method to further improve the algorithm’s solving ability and subsequent connectivity. The optimisation principle is to design the crossover probability as a function related to the fitness function and give full play to the individual fitness value to distinguish the selection probability,

19 $$ p_{c} = \left\{ {\begin{array}{*{20}c} {\frac{{\left( {f^{\prime } - \overline{f} } \right) \cdot \left( {p_{c1} - p_{c2} } \right)}}{{\left( {f_{\max } - \overline{f} } \right)}}} & {f^{\prime } \ge f_{\max } } \\ 0 & {f^{\prime } \le f_{\max } } \\ \end{array} } \right. $$

$p_{c}$ represents the probability of crossover, $p_{c1}$ is the greater probability, $p_{c2}$ is the smaller probability, and $\overline{f}$ represents the average of the population fitness. The essential idea of improving the cross-probability method is to increase the amplitude of the function through the action of the fitness function, thus dynamically distinguishing better individuals according to different individual fitness values. When the current fitness value is lower than the average fitness value, the cross power to the individual is cancelled to ensure that it does not enter the next generation of individuals.

S6: *Mutation.* Similar to the crossover operation, the mutation operation still needs to consider the special coding structure of the original problem. That is, the situation where different occupants are at 0 needs to be considered. If the mutation position occurs on a gene that is 0, it has no practical significance. Therefore, following the coding structure, the nonzero bits are selected to realise the mutation according to the mutation probability to jump out of the optimal local solution that may fall into the random process.

S7: *Termination and output.* When the algorithm converges or reaches the maximum number of iterations, the iteration is terminated. The individual with the largest fitness value in the output population is the optimal solution. Otherwise, enter S3 and continue the iteration.

## Supplementary Information

Supplementary Information.

## Data Availability

All data generated or analysed during this study are included in this article (and its Supplementary Information files).
