# Supplementary material for: Customer demand-driven low-carbon vehicles combined strategy and route optimisation integrated decision
Source: Sci Rep. 2021 Sep 16;11:18483. doi: 10.1038/s41598-021-98028-2 (PMC8445927; doi:10.1038/s41598-021-98028-2)
Supplement: Supplementary file 1 — Supplementary Information. [file 41598_2021_98028_MOESM1_ESM.pdf]

Customer demand-driven low-carbon vehicles combined strategy and route optimisation integrated decision

Hanwen Liu[1*], Xiaobing Liu[2], Sardar MN Islam[3], Xueqiao Yu[4], Qiqi Miao[5], Yapin Chen[6] and Lin Lin[7]

[1,2,5] Dalian University of Technology, School of Economics and Management, Dalian, 116024, China

[3] Victoria University, ISILC, Melbourne, 80309, Australia

[4] China Academy of Railway Sciences Corporate Limited, Transportation & Economics Research Institute, Beijing, 100080, China

[6] Yunnan Normal University, Faculty of Geography, Kunming, 650050, China

[7] CRRC Dalian R&D Co., Ltd., Lvshun, 116052, China

* lucasliu871@gmail.com

Supplementary Table 1 Customer demand of the chemical logistics company

Customer No.	Demand (Ton)	Customer No.	Demand (Ton)
1	27.2	11	28.1
2	31.4	12	30.5
3	13.3	13	32.2
4	25.9	14	17.8
5	19	15	20.5
6	32.1	16	25.3
7	31.7	17	19.2
8	28.4	18	26.6
9	9	19	30
10	11.9	20	21.1

Supplementary Table 2 Symbols and meanings for the joint optimisation model

Symbol	Meaning
Set:	
R	The node set of the transportation network, $R = \{0,1,2,...,n\}$, 0 represents the logistics company service center
U	Connection set of path nodes, $U = \{(i, j) \mid i, j \in R, i \neq j\}$
V	Set of vehicles, $V = \{1,2,...,k\}$,vehicle $k \in V$
M	The number of transport vehicles using traditional energy, and $M = \{1,2,3,...,k\}$, $k \in M$
N	Number of environmental energy vehicles, and $N = \{1,2,3,...,k\}$, $k \in N$
Input parameters:	
E	The number of existing traditional energy vehicles

C_{ev}	Purchase cost of environmental energy vehicles
C_{mo}	Maintenance cost of traditional energy vehicles
C_{me}	Maintenance cost of environmental energy vehicles
C_s	Government subsidies for the purchase of environmental energy vehicles
C_a	Government subsidies for scrapped traditional energy vehicles
C_f	Fuel cost of traditional energy vehicles
C_p	Fuel (electrical) cost of environmental energy vehicles
C_e	Environmental pollution cost
C_b	The cost of replacing batteries in environmental energy vehicles
Q_{ve}	Loading capacity of environmental energy vehicles
Q_{vd}	Loading capacity of traditional energy vehicles
z_{ik}	Cargo loading capacity of transport vehicle k heading to customer i
D_{ij}	Distance from node i to j
CE	Carbon emissions amount of logistics company
CE_q	The amount of carbon allowance given to the company by the regulator
CE_{dv}	Carbon allowance difference
λ	Fuel conversion factor
Decision variables:	
x_{ijk}	$x_{ijk}=1$, choose the k traditional energy vehicle from node i to j, otherwise $x_{ijk}=0$
y_{ijk}	$y_{ijk}=1$, choose the k environmental energy vehicle from node i to j, otherwise $y_{ijk}=0$

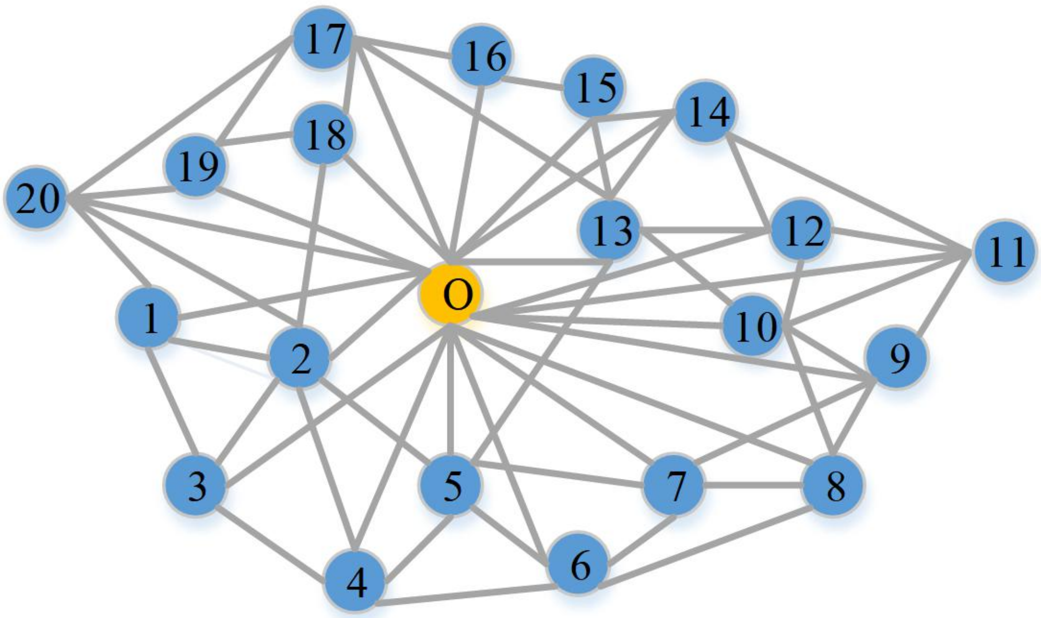

Supplementary Figure 1 Demand network structure diagram. The figure shows that the schematic diagram of the transportation network under customer demand.

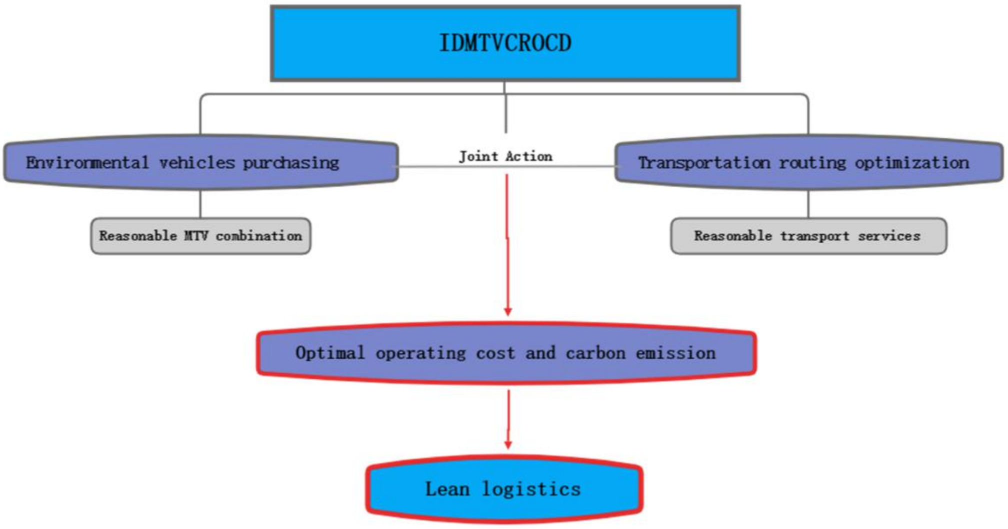

Supplementary Figure 2 The IDMTVCROCD model

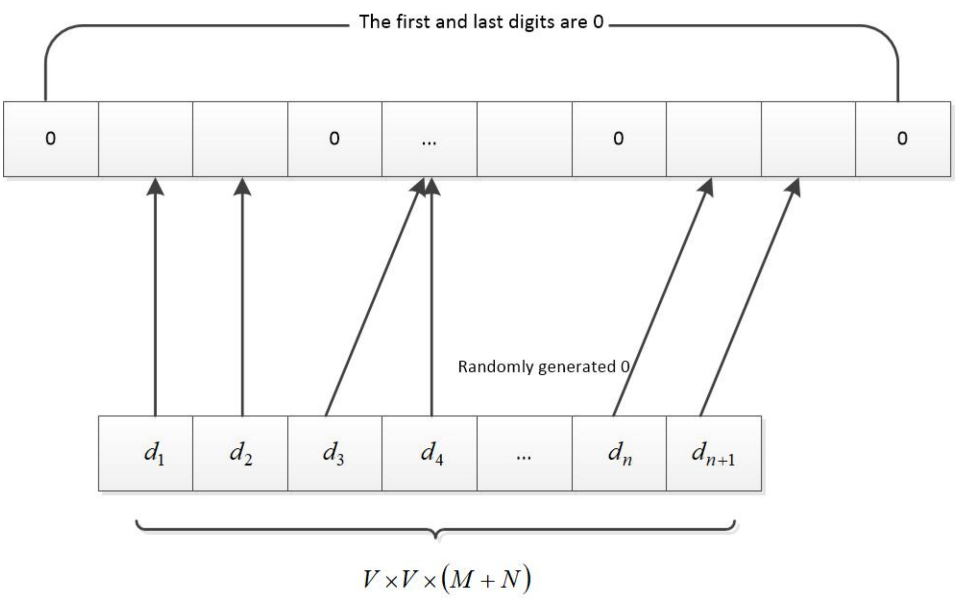

Supplementary Figure 3 The initial chromosome diagram

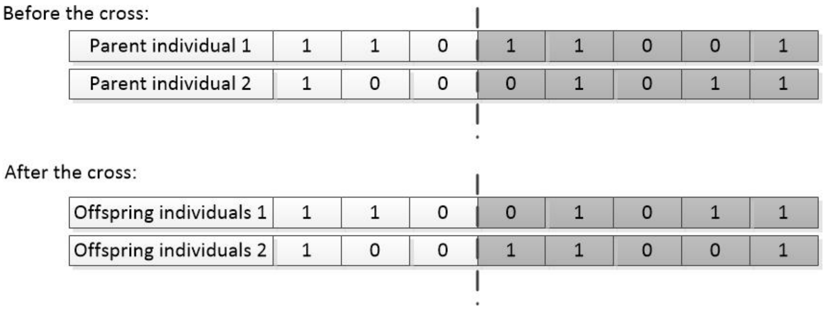

Supplementary Figure 4 Schematic diagram of the traditional single-point crossover method

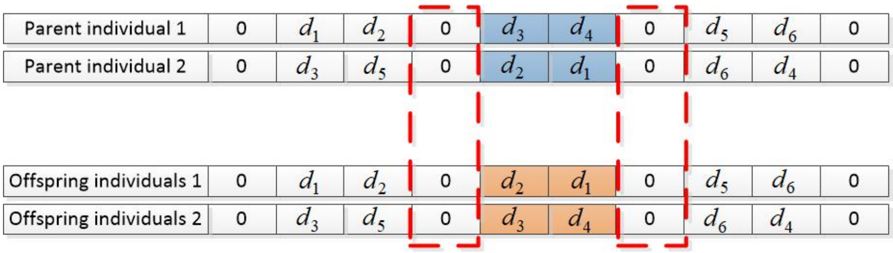

Supplementary Figure 5 Schematic diagram of the improved crossover method
